# A Deep Learning Model Incorporating Knowledge Representation Vectors and Its Application in Diabetes Prediction

**DOI:** 10.1155/2022/7593750

**Published:** 2022-08-12

**Authors:** He Xu, Qunli Zheng, Jingshu Zhu, Zuoling Xie, Haitao Cheng, Peng Li, Yimu Ji

**Affiliations:** ^1^School of Computer Science, Nanjing University of Posts and Telecommunications, Nanjing 210023, China; ^2^Jiangsu High Technology Research Key Laboratory for Wireless Sensor Networks, Jiangsu Province, Nanjing 210023, China; ^3^Institute of High Performance Computing and Big Data, Nanjing University of Posts and Telecommunications, Nanjing 210023, China; ^4^Nanjing Center of HPC China, Nanjing 210023, China; ^5^Jiangsu Research Engineering of HPC and Intelligent Processing, Nanjing University of Posts and Telecommunications, Nanjing 210023, China; ^6^Department of Endocrinology, Zhongda Hospital Southeast University, Nanjing 210009, China

## Abstract

The deep learning methods for various disease prediction tasks have become very effective and even surpass human experts. However, the lack of interpretability and medical expertise limits its clinical application. This paper combines knowledge representation learning and deep learning methods, and a disease prediction model is constructed. The model initially constructs the relationship graph between the physical indicator and the test value based on the normal range of human physical examination index. And the human physical examination index for testing value by knowledge representation learning model is encoded. Then, the patient physical examination data is represented as a vector and input into a deep learning model built with self-attention mechanism and convolutional neural network to implement disease prediction. The experimental results show that the model which is used in diabetes prediction yields an accuracy of 97.18% and the recall of 87.55%, which outperforms other machine learning methods (e.g., lasso, ridge, support vector machine, random forest, and XGBoost). Compared with the best performing random forest method, the recall is increased by 5.34%, respectively. Therefore, it can be concluded that the application of medical knowledge into deep learning through knowledge representation learning can be used in diabetes prediction for the purpose of early detection and assisting diagnosis.

## 1. Introduction

In recent years, with the development of big data and computer technology, intelligent systems based on deep learning method have been used in many fields. Deep learning as an important branch in the field of machine learning has been used in data representations with multiple levels of abstraction through multiprocessing layer models [1]. It has been widely used in the areas of speech recognition [2], image recognition [3, 4], and natural language processing [5]. With the increasing usage of medical equipment and digital recording systems, the amount of patient data is generated and the value of big data is gradually benefiting with the usage of deep learning [6]. Currently, in the medical field, deep learning is mainly used in the research of medical imaging [7, 8] and electronic health record (EHR) [9, 10]. Moreover, physician-level accuracy has been widely achieved in some complex disease diagnosis tasks, such as breast lesion detection [11], diabetes complication prediction [12], and Alzheimer's disease classification [13].

Nevertheless, deep learning-based methods have not yet been widely applied in clinical diagnosis. One of the main factors is due to the black-box feature of deep learning algorithms. The visual or textual explanations provided by deep learning algorithms seem reasonable, but the details of the algorithm's decisions are not clearly exposed [14]. Internals of the model is difficult to grasp for patients or physicians and can contribute to trust issues. Furthermore, it is also against the ethical responsibility of clinicians to leave medical decision-making to black-box systems because it lacks interpretability [15]. In addition, the majority of deep learning models are trained on the basis of data-driven [16] methods, which require that the datasets should be high volume and quality [17]. However, medical datasets are characterized by uncertainty, heterogeneity, time dependence, sparsity, and irregularity [18–20]. These features make the medical datasets that have noisy, missing, and redundant data; thus, it is challenge to guarantee data quality. Besides, security and privacy issues in the healthcare industry restrict the access to healthcare data [21].

Consequently, owing to the black-box feature of deep learning algorithms and the complexity of medical data, it is difficult for using deep learning model to achieve perfect decision-making. However, some research [22] suggests that the knowledge-driven approach can be applied to embed external domain of medical expertise into deep learning models to improve data quality and enhance the interpretability of the models. At present, knowledge-driven approach primarily relies on the building of knowledge graphs [23], such as a knowledge-driven drug reuse approach is proposed in the literature [24], which is based on the constructed comprehensive drug knowledge graphs. Knowledge graphs, as a kind of graph-based data structure, can formally describe real-world matters and their interrelationships [25]. With its huge descriptive power of complex data and better interpretability compared with the traditional methods, it has a promising prospect in smart medical domains [26] and medical knowledge Q&A system [27]. The massive medical knowledge graphs have also been built constantly, such as IBM's Watson Health Knowledge Graph and Shanghai Shuguang Hospital's Knowledge Graph of Chinese Medicine [28]. Intelligent disease diagnosis is aimed at allowing computer machines to learn medical professional knowledge and simulate the analysis of physicians for diagnosis [29], so it is of great research significance to introduce medical professional knowledge into disease diagnosis through knowledge graphs.

However, different diseases are diagnosed differently, and the specialized knowledge has different features. It is worthwhile to consider how the medical knowledge can be widely applied to various disease diagnosis models. In addition, if the appropriate medical knowledge is selected, how to represent this knowledge and combine it rationally with deep learning models remains a challenge. In view of the above problems, this paper selects common physical measurement data as the research object, takes the normal range of medical examination indexes as the professional knowledge, and simulates the process of doctors to make the corresponding diagnosis based on the patient's medical examination data with the normal range of medical examination indicators as the reference in the actual clinical diagnosis. A disease prediction model integrating knowledge representation and deep learning is proposed and applied to diabetes prediction.

The novelty and innovation in this study are summarized as follows.

1. According to the normal range of human physical examination indexes and adopting the knowledge representation learning method, a representation vector of human physical examination index and detection value is constructed. The representation vector can precisely describe the relationships between the physical examination indicators and the detection values, which is suitable for a variety of deep learning models and can increase the interpretability of disease prediction models
2. A deep learning model incorporating a knowledge representation vector is proposed, which employs a constructed representation vector of medical examination indicators and detection values to obtain a relationship matrix of medical examination data. The model associates each medical examination indicator through a self-attention mechanism and utilizes convolutional neural networks for feature extraction for diabetes prediction
3. We compared our model with other machine learning models such as support vector machines and random forests, and the results show that our model is superior to the compared machine learning models in both precision and recall, indicating that the presented model has a better diabetes prediction effect

## 2. Materials and Methods

### 2.1. Deep Learning for Disease Diagnosis

In recent years, a large amount of research work has attempted to apply deep learning to medical diagnosis to assist clinicians and improve the quality of healthcare, for example, medical image classification [30–32], lesion detection [11], and pathology slides [30, 33], as well as electronic health records [34, 35]. Although these researches have yielded valuable results, the lack of interpretability and data quality issues are still key factors limiting their clinical application.

In order to trade off the performance and interpretability of the models, a large number of researchers have researched on interpretable disease diagnosis models [14, 15, 36], focusing on interpreting deep black-box models [37]. For example, Van Molle et al. presented a method which can unravel the black box of convolutional neural networks in the dermatology domain by visualizing the learned feature maps [38]. They concluded that the features which focused on the convolutional neural network were similar to dermatologists for diagnosis. However, the method suffers from the problem that it cannot explain the causal relationship between the features detected by the model and its output, which is not universal. Because it has no specialized knowledge, it is still limited by the quality of the data.

In addition, a number of researches [22, 39–41] have attempted to incorporate domain expertise into deep learning models. Shang et al. constructed a knowledge graph for EHRs to effectively utilize unused information hidden in EHRs [39]. And the semantic rules identified important clinical findings in EHR data. However, the quality of this knowledge graph depends on the amount of data in the EHR. Choi et al. suggested GRAM, a graph-based attention model, which is used to address the data insufficiency and interpretability issues, and supplemented the EHR with hierarchical information inherent to medical ontologies [40]. Ma et al. considered prior medical knowledge in disease risk prediction and successfully introduced a prior medical knowledge into deep learning models using posteriori regularization techniques, and it can be effectively applied to real medical datasets [41].

In the above-mentioned study, the main emphasis was on electronic health records. However, not all patients have complete records, and these records do not exist for patients who may be first-timers in a hospital. Therefore, the broad applicability of these models remains a challenge. To this end, Zou et al. [42] selected relatively easy-to-obtain physical examination data as the subject of their study and used decision trees, random forests, and neural networks to predict diabetes and validated the general applicability of the models in their experiments. However, the models based on machine learning or statistical methods have low performance.

In order to improve the generalization and performance of the model, Alade et al. proposed a feedforward network model for diagnosing diabetes in pregnant women based on expert system and applied it in web applications [43]. Azeez et al. constructed an expert system for disease diagnosis using the Mamdani reasoning method, which can be used to diagnose a variety of diseases [44]. The expert system proposed in literature [43, 44] has wide applicability and greatly improves the accuracy of disease prediction, but it still lacks the support of external professional knowledge.

Combining the advantages and disadvantages of the above studies, this paper comprehensively considers the general applicability of the model and the knowledge of medical expertise. According to the medical examination data of patients, we try to integrate medical expertise and combine with deep learning technology to build a deep learning model incorporating knowledge representation which can be used to assist the diagnosis of diabetes.

### 2.2. Knowledge Representation Learning

**Figure 1:**
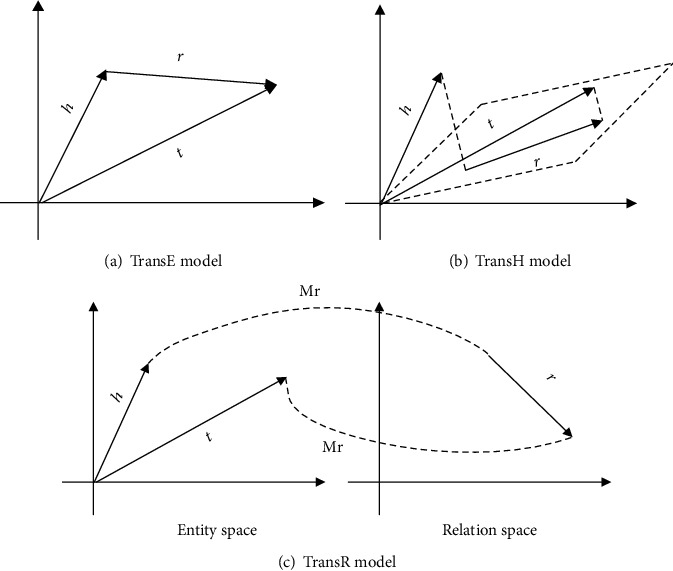
The models of different knowledge representation learning.

Usually, the traditional knowledge graph is represented as triples (*h*, *r*, *t*), where *h* denotes the head entity, *t* denotes the tail entity, and *r* denotes the relationship. Knowledge representation learning [45] represents the research objects (entities and relations) as dense low-dimensional real-valued vectors. Researchers have proposed several knowledge representation models. In this paper, we will introduce the TransE [46], Trans [47], and TransR [48] models which are used in our experiments. The model architecture of the three is shown in Figure 1.

The TransE model [46] uses the vector of relation **l**_*r*_ as a translation between the head entity vector **l**_*h*_ and the tail entity vector **l**_*t*_. Equation (1) shows the relationship of those three vectors.

(1) $$\begin{array}{c} l_{h}+l_{r}\approx l_{t}. \end{array}$$

Its loss function is shown in the following equation:

(2) $$\begin{array}{c} f_{r}\left(h,t\right)={\left|l_{h}+l_{r}-l_{t}\right|}_{L_{1}/L_{2}}. \end{array}$$

That is the *L*_1_ or *L*_2_ distances of the vectors **l**_*h*_ + **l**_*r*_ and **l**_*t*_.

The TransE model has relative parameters, low computational complexity, and high scalability. However, because of the simplicity of the model, the performance of the model is dramatically reduced when dealing with complex relationships. For example, in a one-to-many relationship, suppose there are two triples in the knowledge base, which includes diabetes, complications, and diabetic nephropathy and (, complications, and diabetic foot; if the TransE model is used, it will make the vectors of diabetic nephropathy and diabetic foot become the same, which is obviously inconsistent with the fact. Aiming at solving the shortcomings of TransE in handling complex relationships, the improved TransH and TransR models are proposed, respectively.

The TransH model [45] firstly processes the head entity vector **l**_*h*_ and the tail entity vector **l**_*t*_ along the normal **w**_*r*_ to the hyperplane corresponding to the relation *r*, denoted by **l**_*h*_*r*__ and **l**_*t*_*r*__, respectively. The relationships are shown as follows:

(3) $$\begin{array}{c} l_{h_{r}}=l_{h}-w_{r}^{T}l_{h}w_{r}, \end{array}$$

(4) $$\begin{array}{c} l_{t_{r}}=l_{t}-w_{r}^{T}l_{t}w_{r}. \end{array}$$

Its loss function is shown in the following equation:

(5) $$\begin{array}{c} f_{r}\left(h,t\right)={\left\|l_{h_{r}}+l_{r}-l_{t_{r}}\right\|}_{L_{1}/L_{2}}. \end{array}$$

The TransR model [46] implements the projection of entity vectors onto the subspace of the relation *r* by defining the projection matrix **M**_*r*_ ∈ *R*^(*d* × *k*)^, denoted **l**_*h*_*r*__ and **l**_*t*_*r*__, respectively. The relationship is shown as follows:

(6) $$\begin{array}{c} l_{h_{r}}=l_{h}M_{r},\\ l_{t_{r}}=l_{t}M_{r}. \end{array}$$

Then, it can make **l**_*h*_*r*__ + **l**_*r*_ ≈ **l**_*t*_*r*__, and its loss function is shown in the following equation:

(7) $$\begin{array}{c} f_{r}\left(h,t\right)={\left\|l_{h_{r}}+l_{r}-l_{t_{r}}\right\|}_{L_{1}/L_{2}}., \end{array}$$

## 3. Model Architecture

In this paper, a disease prediction model fusing knowledge representation and deep learning is proposed, which is aimed at simulating the process of disease diagnosis by physicians based on the patient's physical examination data and the known normal range of physical examination indexes. The method obtains a matrix representation of patient physical examination data which is input into a deep learning model to get the result of disease prediction.

**Figure 2:**
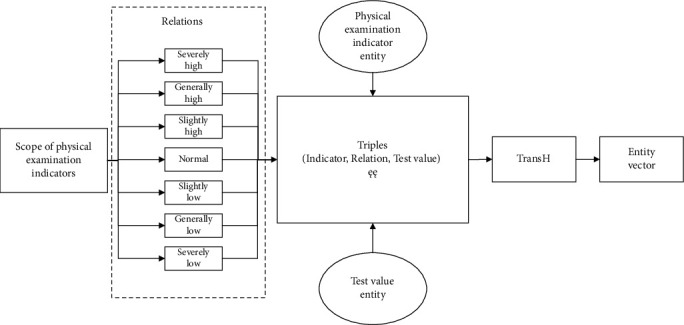
Physical examination indicator entity and test value entity to vector.

The architecture of the deep learning model incorporating the knowledge representation vector is shown in Figure 2. It is mainly divided into the following three parts:

1. According to the normal range of the test value of the physical examination indicator, the relationship between the physical examination indicator and the detection value is constructed, and then, the knowledge representation learning model is used to acquire the representation vector of the physical examination indicator and the detection value
2. We obtain the physical examination data of patients, acquire the relationship vectors between all physical examination indicators and corresponding test values according to the representation vectors of physical examination indicators and test values in (1), and splice them into a relationship matrix
3. The relationship matrix is input to the classifier constructed by the self-attention mechanism (self-attention) and convolutional neural network (CNN) to obtain the prediction results of diabetes

In this paper, the proposed model is referred to TH-SAC. The choice of SAC model was made by comparing various models through reading literature and experiments. This paper first tries classical machine learning methods such as logistic regression and random forest, but these methods have been widely used in disease prediction, and it is difficult to improve the prediction effect. Therefore, we began to try to use the method of deep learning. Firstly, we got the vector representation of physical examination index values through knowledge representation learning. Because a single physical examination indicator cannot fully reflect the disease status, different indicators will affect each other. Through reading literature, we know that self-attention can obtain global information, so we choose the self-attention mechanism to calculate the interaction between different indicators. However, self-attention calculations alone were used to extract features that accurately reflected disease. In this regard, CNN extraction is introduced for feature extraction on the basis of self-attention. Of course, we also had tried DNN, Bi-LSTM, and other models, as well as self-attention and CNN alone, but the effect was not good when we evaluated the accuracy, recall rate, F1 value, and so on; at last, we finally chose SAC model.

### 3.1. Representation Vector of Physical Examination Indicators and Detection Values

In the actual clinical diagnosis of diseases, physicians often make judgments by combining the patient's physical examination data and existing physical examination knowledge. For example, the normal range of blood glucose values in the clinical diagnosis of diabetes is 3.9-6.1 mmol/L. When the blood glucose value is greater than 7.0 mmol/L, it is considered as a possibility of diabetes mellitus [49]. As shown in Figure 2, this paper considers embedding such medical expertise in the model, namely, the normal range of physical examination indicators. Firstly, according to the normal range of physical examination indicators defined in medical science and the advice of medical experts, the values of relevant physical examination indicators are divided into seven ranges: severely low, generally low, slightly low, normal, slightly high, generally high, and seriously high. The measured value of each physical examination index corresponds to a range; that is, there is a relationship between the physical examination index and the measured value. For example, if the normal range of triglycerides is 0.45-1.81 mmol/L, the relationship between triglycerides and 0.45.mmol/L is normal. This relationship can be expressed in triplet form (triglyceride, normal, 0.45.mmol/L), which is the basic form of the knowledge graph. Through this method, all relevant physical examination indicators and their corresponding test values are expressed in the form of the above triplet, and then, the physical examination indicators and the corresponding test values are expressed in vector form through knowledge representation learning method.

Since it exists complex one-to-many and many-to-one relationships between the physical examination indexes and the corresponding test values, the TransH model chosen in this paper is more suitable with this kind of relational representation. Therefore, after converting the knowledge of the physical examination into the form of triples, the representation vector is obtained using the TransH model. The model uses the translation vector and the normal vector of the hyperplane to represent the relation *r*. The projection vectors of the entity vector and the hyperplane which is called as the relation *r* are calculated according to equations (3) and (4). Then, according to equation (5), the low-dimensional dense representation entity vector of the physical examination index and the test value is acquired.

### 3.2. Relationship Vector of Physical Examination Indicators and Detection Values

After getting the representation vector of the medical examination knowledge, in order to reflect the relationship between the medical examination indicators and their corresponding test values in the model, we use the difference between the entity vector of each medical examination indicator and its corresponding test value to represent the relationship. Based on the basic idea of knowledge representation learning model, **l**_*h*_ + **l**_*r*_ ≈ **l**_*t*_, the relationship between the entity vector of physical examination indicator and its corresponding entity vector of detection value is represented by the difference, as shown in the following equation:

(8) $$\begin{array}{c} e_{r}=e_{v}-e_{c}, \end{array}$$

where **e**_*v*_ is the entity vector of test values, **e**_*c*_ is the entity vector of physical indicators, and **e**_*r*_ is the corresponding relationship vector between **e**_*v*_ and **e**_*c*_.

For example, the relationship between the physical vector **e**_fasting blood glucose_ of fasting blood glucose and the physical vector **e**_7.1mmol/L_ of the test value (7.1 mmol/L) is expressed as **e**_7.1mmol/L_ − **e**_fasting blood glucose_. The relationship vectors **e**_*r*_^*i*^ between all the physical indicators and their corresponding test values are combined to form the relationship matrix between the physical indicators and the test values of one person, as shown in the following formula:

(9) $$\begin{array}{c} E^{k\times m}=\left[e_{r}^{1},e_{r}^{2},e_{r}^{3},\cdots ,e_{r}^{m}\right]. \end{array}$$

Among them, *k* is the dimension of the entity vector, and *m* is the number of physical examination indicators.

### 3.3. SAC Classifier

**Figure 3:**
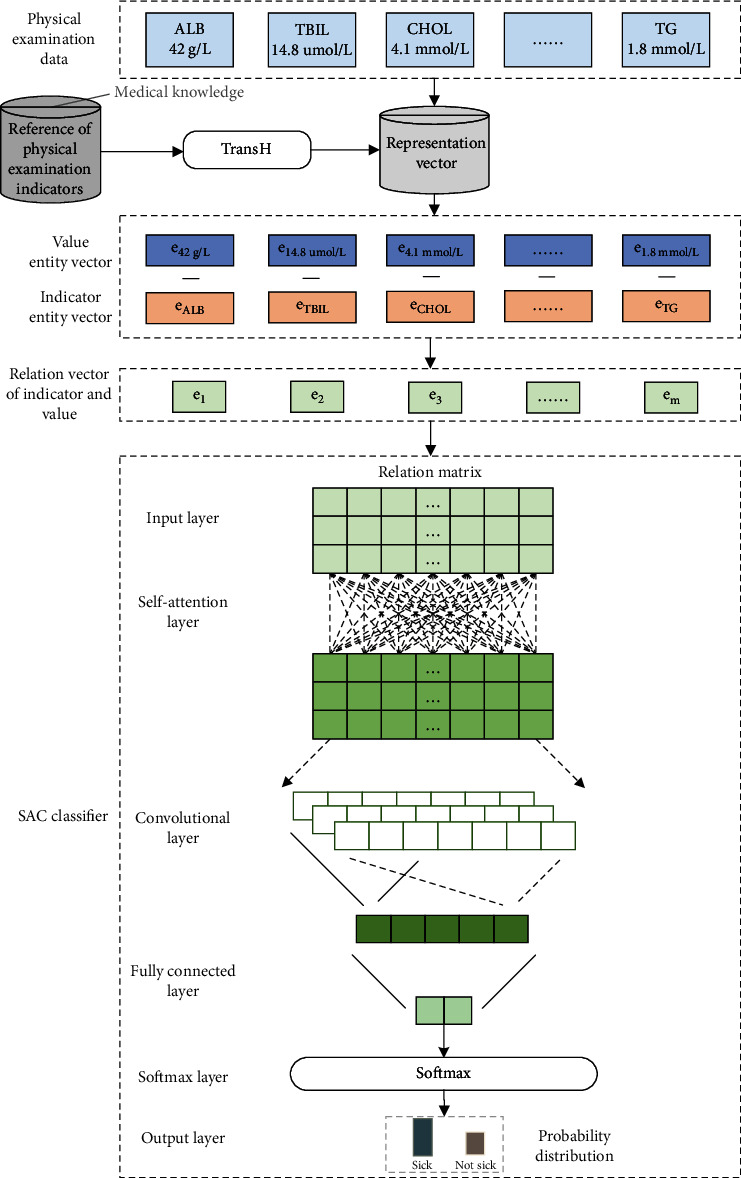
TH-SAC: architecture diagram of a disease prediction model integrating knowledge representation and deep learning.

**Figure 4:**
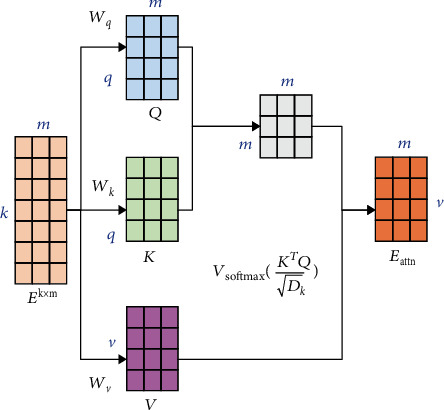
Self-attention mechanism

The SAC classifier is key part of the TH-SAC model in Figure 3 and consists of the following layers:

1. Input layer: the relationship matrix **E**^*k*×*m*^ obtained by splicing the relationship vectors between all the physical examination indicators and the corresponding detection values is the input of the classifier
2. Self-attention layer: since each medical examination index is interrelated, the relationship matrix **E**^*k*×*m*^ is further input into the self-attention layer, so that each medical examination index can get global information, which is in line with the current medical diagnosis experience. In this paper, the number of layers adopted for the self-attentive layer is 2 in our attention mechanism. As shown in Figure 4, in the attention layer, the following three weights **w**_*q*_ ∈ **R**^*q*×*k*^, **w**_*k*_ ∈ **R**^*q*×*k*^, and **w**_*v*_ ∈ **R**^*v*×*k*^ are first defined, and each relation vector **e**_*r*_^*i*^ is linearly mapped into three different spaces according to equations (10)–(12) to get the query vector **q**_*i*_, the key vector **k**_*i*_, and the value vector **v**_*i*_:

(10) $$\begin{array}{c} q_{i}=w_{q}e_{r}^{i}, \end{array}$$

(11) $$\begin{array}{c} k_{i}=w_{k}e_{r}^{i}, \end{array}$$

(12) $$\begin{array}{c} v_{i}=w_{v}e_{r}^{i} \end{array}$$

For each query vector **q**_*i*_, we can calculate the output vector **e**_attn_ according to the following equation:

(13) $$\begin{array}{c} e_{\text{attn}}^{i}=\overset{m}{\underset{j=1}{\sum }}a_{ij}v_{i}, \end{array}$$

where *a*_*ij*_ denotes the weight of the *i*th output concern to the *j*th input, which is calculated from the following equation:

(14) $$\begin{array}{c} a_{ij}=\text{softmax}\left(s\left(k_{j},q_{i}\right)\right),\\ s\left(k_{j},q_{i}\right)=\frac{k_{j}^{T}q_{i}}{\sqrt{D_{k}}}, \end{array}$$

where softmax(∙) is a function normalized by columns and *D*_*k*_ is the dimension of **q**_*i*_.

In order to simultaneously calculate the output vector corresponding to each relation vector in the relation matrix **E**^*k*×*m*^, the query vector **q**_*i*_, the key vector **k**_*i*_, and the value vector **v**_*i*_ can be merged into the query matrix **Q**, the key matrix **K**, and the value matrix **V**, respectively. Then, the output matrix of the attention layer is obtained according to the following equation:

(15) $$\begin{array}{c} E_{\text{attn}}=V\text{softmax}\left(\frac{K^{T}Q}{\sqrt{D_{k}}}\right). \end{array}$$

(3) Convolutional layer: after acquiring the global information through the self-attention layer, the output matrix **E**_attn_ of the self-attention layer is input to the convolutional neural network in purpose of mining the information in the relationship matrix using deep learning model. Suppose **W**^*f*^ ∈ *R*^*h*×*d*^, where *h* is the filter window size and *d* denotes the dimensionality of the input vector. For the local features **e**_attn_^*i*:*i*+*h*−1^ of the input from row *i* to row *i* + *k* − 1, the *i*th eigenvalue of the feature submatrix extracted by the convolutional filter is expressed as

(16) $$\begin{array}{c} c_{i}=f\left(w^{f}\cdot e_{\text{attn}}^{i:i+h-1}+b\right), \end{array}$$

where *f*(·) is the nonlinear activation function relu(·) and *b* is the bias value. Thus, the local feature matrix of the output matrix **E**_attn_ obtained from the attention layer is

(17) $$\begin{array}{c} C=\left[c_{1},c_{2},c_{3},\cdots ,c_{m-h+1}\right] \end{array}$$

Subsequently, a maximum pooling operation is performed on each feature mapping, i.e.,

(18) $$\begin{array}{c} \hat{c}=\max\left\{C\right\}. \end{array}$$

Then, the final representation vector of the medical examination data is obtained as shown in the following equation:

(19) $$\begin{array}{c} Z_{tj}=\left[{\hat{c}}_{1},{\hat{c}}_{2},{\hat{c}}_{3},\cdots ,{\hat{c}}_{n}\right]. \end{array}$$

(4) Fully connected layer and softmax layer: the representation vector of medical examination data is transformed by the fully connected layer to obtain the score vector **s** which can be used to predict diabetes. The quantity of hidden units in the fully connected layer is 2, i.e., diabetic and nondiabetic. Finally, the score vector s is input to the softmax layer which can transform to a conditional probability distribution:

(20) $$\begin{array}{c} p_{i}\left(s\right)=\frac{\exp\left(s_{i}\right)}{\sum_{j=1}^{2}\exp\left(s_{j}\right)},\;i=1,2 \end{array}$$

The whole model uses a crossentropy loss function to measure the gap between the predicted probability distribution of diabetes and the real probability distribution, and the parameters of the model are trained and updated by a back-propagation algorithm. The loss function is denoted as

(21) $$\begin{array}{c} \text{Loss}=-\frac{1}{N}\underset{i}{\sum }\left[y_{i}\cdot \log\left(p_{i}\right)+\left(1-y_{i}\right)\cdot \log\left(1-p_{i}\right)\right], \end{array}$$

where *N* represents the number of samples and *y*_*i*_ represents the true label of sample *i* and with disease is marked as 1 and no disease is marked as 0.

## 4. Experiment and Results

### 4.1. Experimental Data

The data that are used in the experiments are derived from the reference range of the detection values of diabetes physical examination indexes provided by Zhongda Hospital, China. The procedure of this study was approved by the Research Ethics Committee of Zhongda Hospital affiliated to Southeast University (Approval no. of Ethics Committee: 2019ZDSYLL199-P01), China.  Table 1 shows the reference ranges of the test values of medical indicators. Based on this physical examination knowledge, a total of 5518 related entities, 7 types of relational entities (severely low, generally low, slightly low, normal, slightly high, generally high, and severely high), and 9410 ternary relationships are established. The types of entities and their quantities are shown in Table 2, and the types of relationships and their numbers are shown in Table 3. As it is impossible to predict the threshold value of each physical test index in practice, the entities with detection values greater than (less than) the maximum (minimum) value set in the experiment are uniformly treated as abnormally high <HIGHEST> entities (abnormally low <LOWEST> entities). In addition, all missing value items were replaced by the unknown entity <UNK>.

The physical examination data of diabetic patients provided by a large company is adopted, which contains 11 physical examination indicators, such as serum alanine aminotransferase, serum aspartate aminotransferase, and albumin, with a total number of 48887. And the training set accounts for 80%, and the test set accounts for 20%, as shown in Table 4.

### 4.2. Experimental Setup

The deep learning framework, PyTorch, and the knowledge representation learning framework, OpenKE, are primarily utilized in the experiments. The specific parameter settings of model are shown in Table 5. In this paper, the hyperparameter values selected in the model are optimized by grid search algorithm, and the accidental selection of the hyperparameter values is prevented by the cross-validation of fivefold.

### 4.3. Evaluation Indicators

In this paper, accuracy, recall and F1_score are adopted. Mean rank (MR) and Hit@10 are chosen as the evaluation metrics of the knowledge representation model. In addition, in order to improve the credibility, the experimental results are verified by the fivefold cross-validation method.

#### 4.3.1. Mean Rank

When evaluating the performance of the knowledge representation learning model, each triple(*h*, *r*, *t*)is evaluated, the head entity is removed and replaced with other entities in the knowledge base in turn, and the wrong triple entity (*h*, *r*, *t*) is constructed. The similarity of head and tail entities using the relation function *f*_*r*_(*h*, *t*) is calculated. After getting the similarity from all the triples (including the correct triples and the incorrect triples), the triples are sorted in an ascending order. The average value of all correct triple ranking positions is the MR. For better knowledge graph representation, the score of the correct triad will be smaller than the score of the incorrect triad and will be ranked more highly. Therefore, the smaller the MR value is, the better the knowledge mapping representation vector is. Specifically, MR is shown in the following equation:

(22) $$\begin{array}{c} \text{MR}=\frac{1}{N_{T}}\overset{N_{T}}{\underset{i=1}{\sum }}{\mathrm{rank}}_{i}, \end{array}$$

where *N*_*T*_ denotes the number of correct triples and rank_*i*_ represents the ranking of the correct triples.

#### 4.3.2. Hit@10

The ratio of the number of correct triples contained in the top 10 of the above ranking to the total amount of correct triples is the Hit@10 value. Therefore, the larger the Hit@10 value is, the better the knowledge graph representation vector is. Specifically, as shown in the following formula,

(23) $$\begin{array}{c} \text{Hit}@10=\frac{N_{T}^{\mathrm{rank}\leq 10}}{N_{T}}\times 100\%, \end{array}$$

where *N*_*T*_^rank≤10^ represents the number of correct triples in the top ten.

### 4.4. Analysis of Experimental Results

#### 4.4.1. Comparative Analysis of Knowledge Representation Models

At first, the performance of different knowledge representation models is analyzed, and the results are illustrated in Tables 6 and 7. As shown in Table 6, from the comprehensive MR metrics and Hit@10 metrics, the TransH model performs the best effect of knowledge representation. This demonstrates that TransH can better deal with the complex relationships of “one to many” and “many to one” between physical examinations and detection values, which makes up for the deficiency of TransE. Although the TransR model takes into account these complex relationships, there are only similar relationships between the physical examination and the test value, such as high and low, and the different relationships focus on the similar properties of the entities, so the TransR model does not perform well in knowledge representation.

In addition, from Table 7, we can see that the TransH model outperforms both the TransE model and the TransR model by 0.07% and 0.15% in accuracy and 0.29% and 0.56% in recall, respectively. This further indicates that the representation of the TransH model is more rational for the triples constructed in this paper based on medical examination knowledge, which also makes the performance of prediction model better. The text continues here (Figure 3 and Table 2).

#### 4.4.2. Comparative Analysis

For the purpose of verifying the advantages of the proposed TH-SAC model for the diabetes prediction task, some relevant diabetes prediction models are selected for experiments. The TH-SAC model is used to represent the medical examination data as vectors through knowledge representation learning, and deep learning approach is used for prediction. In this paper, the traditional machine learning methods that work well on diabetes prediction tasks and deep neural networks (DNN) are selected for comparisons, and the results are illustrated in Table 8. Compared with the most effective random forest methods in machine learning, it can be seen that the TH-SAC model has been improved by 0.81% and 5.34% in accuracy and recall, respectively. This is because our model is based on deep learning approach and adopts a self-attention architecture, which is better at mining effective information from complex and high-dimensional medical examination data. Compared with that of DNN method, the accuracy and recall rate are improved by 6.97% and 28.83%, respectively. The results show that the method of representing medical examination data as vectors through knowledge representation learning is more superior to simply employing detection values. The embedded external knowledge not only improves the interpretability of the model but also enhances the performance of the model.

Moreover, the classifier used in the TH-SAC model is designed and implemented by integrating self-attention and convolutional neural networks (CNN). Therefore, we select the following methods for comparative experiments: self-attention and CNN are used alone, and the results are shown in Table 8. It can be seen that the SAC classifier has better performance in terms of accuracy and recall. This is because the SAC classifier is able to integrate the local features with their corresponding global dependencies, which provides more superior performance than that of only self-attention or CNN alone.

#### 4.4.3. SMOTE Resampling Analysis

**Figure 5:**
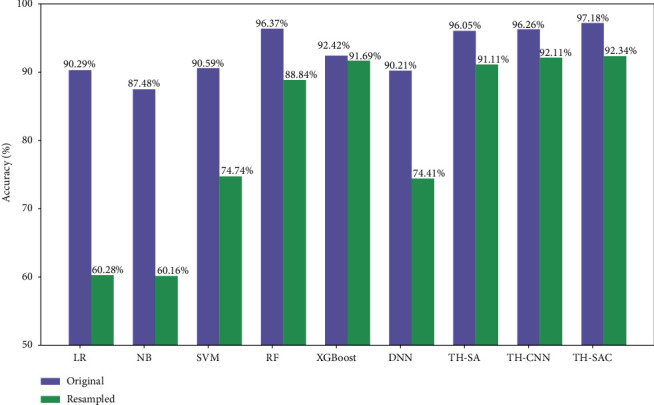
Comparison of accuracy of each model before and after resampling.

**Figure 6:**
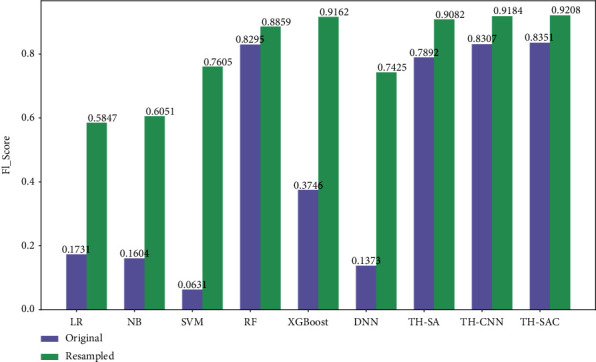
Comparison of F1 values for each model before and after resampling.

As shown in Table 4, the number of negative samples in the dataset used is much larger than the number of positive samples, and there exists the problem of unbalanced distribution. However, the degree of imbalance in the dataset can affect the accuracy of the model as well as the generalization ability [50]. In this paper, the SMOTE [51] method is adopted to address the problem of imbalanced distribution of the dataset. As shown in Table 9, the distribution of positive and negative samples in the dataset after resampling with the SMOTE method reaches a balanced state. After SMOTE resampling, the above prediction models were experimented again and compared. The experimental results are shown in Figures 5 and 6 . The accuracy of all models on the balanced dataset after resampling has decreased, and the F1 values has increased. The results indicated that more illnesses were predicted to be nonillnesses before resampling, while more nonillnesses were predicted to be illnesses after resampling. Additionally, comparing the performance of all models on the resampled datasets, the model proposed in this paper still outperforms the other models in terms of accuracy and F1 values. Moreover, it is less affected by the datasets and the accuracy of the model does not fluctuate significantly. This further proves the applicability as well as the effectiveness of the TH-SAC model.

#### 4.4.4. Comparative Analysis of Knowledge Representation and Embedding Representation

In order to verify the effectiveness of incorporating external medical examination knowledge, this paper compares the embedding representation and knowledge representation of medical examination index entities and detection value entities. The embedding representation refers to the one-hot encoding of all entities and then multiplying them with a weight matrix. The comparison results are shown in Table 10; it can be seen that the knowledge representation significantly outperforms the random representation model in terms of prediction performance. This indicates that the entity vector constructed in this paper by the relationship between the physical examination index and the detection value plays a good role.

**Figure 7:**
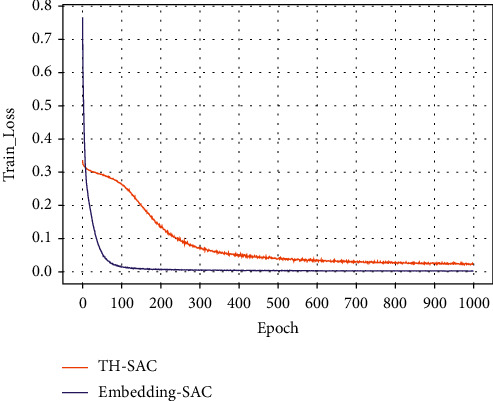
Training loss values of the original dataset.

**Figure 8:**
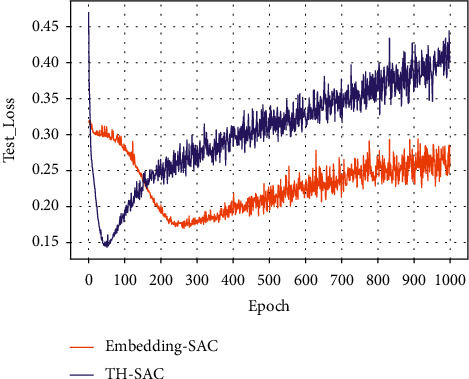
The test loss value of the original data set.

**Figure 9:**
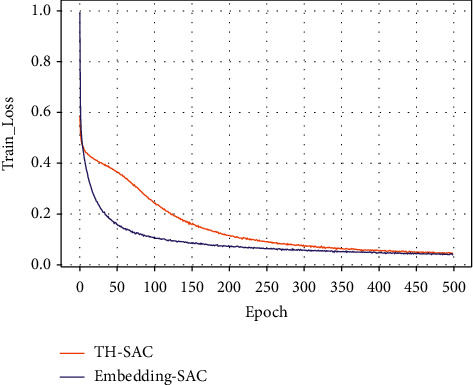
Training loss values for resampled dataset.

**Figure 10:**
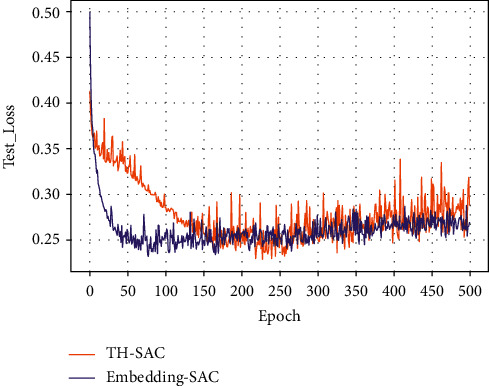
Test loss value for resampling dataset.

Figures 7–10 present the training loss values and test loss values of the two models on the original and resampled datasets. It can be seen that the loss values of the TH-SAC model in the initial training are smaller than those of the models without the introduction of knowledge, indicating that the vectors obtained by training the normal range of the medical examination indexes can provide a better representation of the relationship between the medical examination indexes and their corresponding detection values. In addition, as shown in Figures 8–10, the model with the embedded representation has the risk of overfitting.

#### 4.4.5. Comparative Analysis of Representation Vectors with Different Dimensions

**Figure 11:**
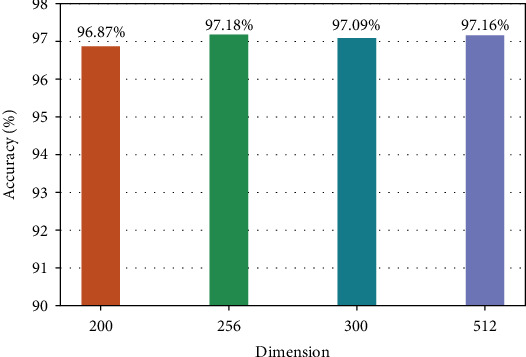
Accuracy of representation vectors in different dimensions.

**Figure 12:**
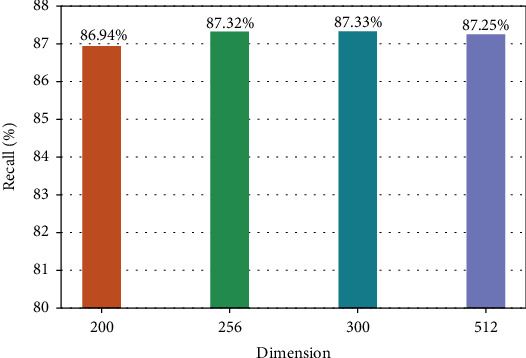
Recall of representation vectors in different dimensions.

In the process of knowledge representation learning, if the vector dimension is too small or too large, there exists a risk of overfitting. In order to select the optimal dimension of the representation vector, the number of four dimensions of 200, 256, 300, and 512 is selected in our experiments, and the results are shown in Figures 11 and 12.

From Figures 11to 12, it can be seen that the accuracy and recall are lower in the lower 200-dimensional representation vector because the information contained is not comprehensive. However, the higher the dimension is, the more complex the model parameters are, and the longer the training time is. Therefore, considering the accuracy, recall, and complexity of the model parameters, we selected the dimension number of the representation vector which is 256 in this paper.

#### 4.4.6. Self-Attention Mechanism Weight Visualization

**Figure 13:**
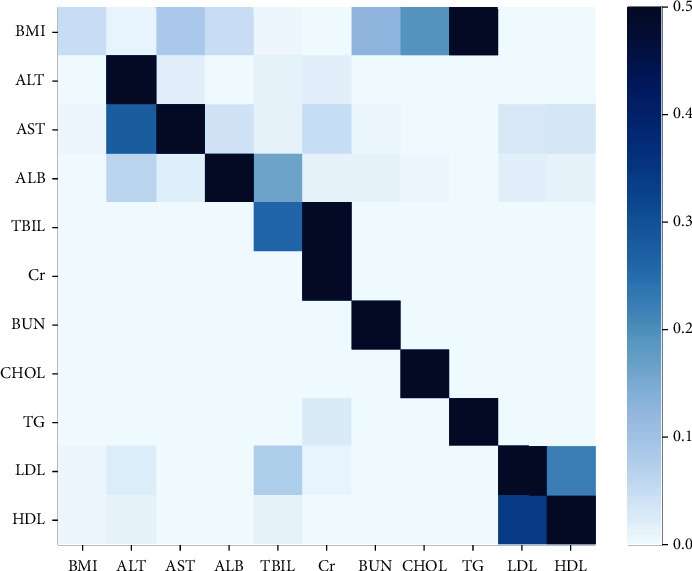
Visualization of the weight of the self-attentive layer.

As shown in Figure 13 and Table 11, the weights of the self-attention layer in our model are visualized. It can be seen that there is a strong correlation between low-density lipoprotein (LDL) and high-density lipoprotein (HDL) and BMI with total cholesterol and triglycerides, which is consistent with the medical expertise. It also indicated that the model proposed in this paper has the interpretability.

## 5. Conclusions

Deep learning generally has problems in the medical field, such as insufficient data, low quality, and lack of interpretability of models. In this paper, a disease prediction model combining knowledge representation and deep learning is proposed and applied in the field of diabetes. According to the relationship between physical examination index and test value, the vector representation of physical examination knowledge entity is constructed through the TransH model, and then, the relationship matrix of patient physical examination data is obtained. Then feature extraction was carried out through the constructed self-attention mechanism and convolutional neural network, and a deep learning model for disease prediction was designed and implemented. In the experiment, the accuracy rate and recall rate of the model in this paper were 97.18% and 87.55%, respectively, which were better than those of the traditional machine learning method and the deep learning method without introducing knowledge representation. Therefore, the medical knowledge introduced in this paper improves the validity and efficiency of the model to a certain extent.

However, there are still some limitations to our approach. In this paper, the range of physical examination index values is divided according to the experience of medical experts, except the normal range. Moreover, the accuracy of prediction depends to some extent on the accuracy of range division, so whether the range division is optimal remains to be further studied. In addition, the knowledge of physical examination used in this paper is incomplete and does not take into account the relationship between the normal range of physical examination indicators and age and sex. And the patient's related symptoms and the actual clinical diagnosis are different. In the next step, we will try to introduce the above relationship to improve the disease prediction model and build a computer-aided diagnosis system.

## Figures and Tables

**Table 1:** The reference range of detection value of some physical examination indicators.

Medical examination indicator	Reference range
Serum alanine aminotransferase	9-50 IU/L
Serum aspartate aminotransferase	15-40 IU/L
Albumin	40.0–55.0 g/L
Total bilirubin	2.0–20.0 *μ*mol/L
Blood urea nitrogen	3.6-9.5 mmol/L
Total cholesterol	2.86-6.10 mmol/L
Triglycerides	0.45-1.81 mmol/L
Low-density lipoprotein	0.00-3.37 mmol/L
High-density lipoprotein	1.16-1.42 mmol/L
……	……

**Table 2:** Entity type and quantity.

Type of entity	Example	Number of entities
Medical examination indicator	Triglycerides	16
Detection value	1.62 mmol/L	5499
Abnormally high	<HIGHEST>	1
Abnormally low	<LOWEST>	1
Unknown	<UNK>	1
Medical examination indicator	Triglycerides	16

**Table 3:** Relationship type and quantity.

Relationship types	Number of entities
Severely low	337
Generally low	343
Slightly low	457
Normal	1558
Slightly high	2663
Generally high	2005
Severely high	2017

**Table 4:** The distribution of physical examination dataset.

Disease label	Training set	Test set
Diabetes	3815	954
Nondiabetes	35924	8824
Total	39109	9778

**Table 5:** Model parameter settings.

Parameter	Value
Optimizer	Adam
Batch_size	32
Epoch	100
Dropout	0.5
Learning rate	0.0002
Entity vector dimension of physical examination data	256
Size of convolution filter window	2, 3, 4
Number of convolution filters per window size	100
Number of layers of self-attention	2

**Table 6:** MR and Hit@10 of different knowledge representation model.

Model	MR	Hit@10 (%)
TransE	623.0	44.9
TransH	711.6	47.9
TransR	897.8	19.0

**Table 7:** Accuracy and Recall Rates of Different Knowledge Representation Models.

Model	Accuracy (%)	Recall (%)	F1
TransE-SAC	97.11	87.16	0.8300
TH-SAC	97.18	87.55	0.8351
TransR-SAC	97.03	86.89	0.8295

**Table 8:** Accuracy and recall rates of different diabetes prediction models.

Model	Accuracy (%)	Recall (%)	F1
LR	90.29	49.9	0.1731
SVM	90.59	51.60	0.0631
NB	87.48	53.94	0.1604
RF	96.37	82.11	0.8295
XGBoost	92.42	61.64	0.3746
DNN	90.21	58.62	0.1373
TH-SA	96.05	86.15	0.7892
TH-CNN	96.26	87.03	0.8307
TH-SAC	97.18	87.55	0.8351

**Table 9:** Distribution of sampled physical examination dataset.

Disease label	Training set	Test set
Diabetes	35294	8824
Nondiabetes	35924	8824
Total	70588	17648

**Table 10:** Accuracy and recall rates of different diabetes prediction models.

Model	Accuracy (%)	Recall (%)	F1
Embedding-SAC	96.98	86.32	0.8253
TH-SAC	97.18	87.32	0.8351

**Table 11:** The numerical values of the weight of the self-attentive layer.

	BMI	ALT	AST	ALB	TBIL	Cr	BUN	CHOL	TG	LDL	HDL
BMI	0.044682	0.0063637	0.082328	0.043529	0.0024994	0.0017869	0.12636	0.18994	0.50183	0.00024223	0.00043629
ALT	4.254*e*-05	0.95567	0.013733	0.0014261	0.012931	0.014677	8.8271*e*-05	0.0003062	6.7363*e*-06	0.00067861	0.00044125
AST	0.0024338	0.27391	0.57118	0.036222	0.0088331	0.045473	0.0044836	0.00071565	1.6739*e*-05	0.025368	0.031365
ALB	0.001296	0.06182	0.17392	0.70461	0.16075	0.013515	0.0104	0.0032933	0.00039018	0.014664	0.011861
TBIL	1.9193*e*-08	2.5626*e*-07	4.1367*e*-08	2.6728*e*-07	0.25978	0.74022	2.3709*e*-07	8.6627*e*-07	4.7918*e*-06	5.7109*e*-07	1.6839*e*-07
Cr	8.3536*e*-16	9.8131*e*-14	5.6186*e*-16	9.2363*e*-14	2.0324*e*-12	1.0	9.2502*e*-14	8.219*e*-14	4.5131*e*-13	3.925*e*-14	4.0831*e*-14
BUN	2.4488*e*-05	3.7696*e*-05	3.0095*e*-05	0.000663	6.1397*e*-05	2.97775*e*-05	0.99901	3.3287*e*-06	3.4574*e*-06	5.81*e*-05	8.1024*e*-05
CHOL	4.7872*e*-07	2.9578*e*-08	2.7738*e*-08	7.6755*e*-07	8.7449*e*-08	1.0327*e*-09	1.8206*e*-07	0.99996	1.4392*e*-07	4.8578*e*-06	3.181*e*-05
TG	3.7574*e*-07	7.568*e*-09	5.1691*e*-10	1.1795*e*-06	2.7954*e*-06	0.023429	2.0787*e*-05	0.00045578	0.97609	4.4345*e*-08	1.37772*e*-07
LDL	0.0024032	0.018078	0.001621	0.0015858	0.077337	0.0070292	0.00026765	0.00030051	9.7969*e*-05	0.66945	0.22183
HDL	0.0038618	0.0082498	0.0018781	0.0011091	0.011362	0.00010898	0.00043675	0.00049594	8.7666*e*-05	0.34158	0.63083

## Data Availability

The data presented in this study are available from the corresponding authors on reasonable request.
